# LCN2 as a Potential Diagnostic Biomarker for Ulcerative Colitis-Associated Carcinogenesis Related to Disease Duration

**DOI:** 10.3389/fonc.2021.793760

**Published:** 2022-01-17

**Authors:** Fushun Kou, Yuan Cheng, Lei Shi, Jiajing Liu, Yuyue Liu, Rui Shi, Guiying Peng, Junxiang Li

**Affiliations:** ^1^ Graduate School, Beijing University of Chinese Medicine, Beijing, China; ^2^ Gastroenterology Department, Dongfang Hospital, Beijing University of Chinese Medicine, Beijing, China; ^3^ School of Life Sciences, Beijing University of Chinese Medicine, Beijing, China

**Keywords:** LCN2, ulcerative colitis, long-duration disease, ulcerative colitis-associated carcinogenesis, colorectal cancer

## Abstract

**Background:**

Patients with long-duration ulcerative colitis (UC) had a higher risk of developing ulcerative colitis-associated carcinogenesis (UCAC) when compared to those with short-duration UC. This study aimed to discover the biomarker for cancer surveillance related to disease duration.

**Methods:**

The microarrays were divided into short-duration (<10 years) UC, long-duration (≥10 years) UC, UCAC, and normal groups in the Gene Expression Omnibus (GEO) datasets. Differentially expressed genes (DEGs) of GEO and the hub genes of the selected weighted gene co-expression network analysis (WGCNA) were intersected to obtain the overlapping genes. Among these genes, the key gene was identified by using the protein–protein interaction (PPI) network, Gene Ontology (GO), Kyoto Encyclopedia of Genes and Genomes (KEGG) analysis, the cytoHubba of Cytoscape, and the expression levels. Also, immunofluorescence of human colonic mucosa and animal experiment were used to validate the expression trend of the key gene in the progress of UC developing into UCAC.

**Results:**

Lipocalin-2 (LCN2) was more relevant with disease duration of UC and significantly negatively correlated with the risk of UCAC. The expression level of LCN2 in short-duration UC was higher than that of long-duration UC (*P* < 0.01), long-duration UC was higher than that of UCAC (*P* = 0.001), and UC and UCAC were all higher than that of the normal (*P* < 0.001). We then discovered that the expression trend of LCN2 in blood and stool samples was consistent with that in colorectal mucosa.

**Conclusion:**

The research indicates that LCN2 could be a novel biomarker to evaluate cancer surveillance related to disease duration of developing UC into UCAC. Compared with that of blood samples, stool detection of LCN2 may have more advantages for diagnosis value of early stage of UCAC as a complement to colonoscopy surveillance.

## Introduction

Ulcerative colitis (UC) is a group of chronic idiopathic inflammatory diseases of the gastrointestinal tract and characterized by symptoms that evolve in a relapsing and remitting manner. UC has become a more common disease in many parts of the world. The prevalence of UC varies considerably across different countries and is higher in Europe (505/100,000 in Norway) and North America (286/100,000 in the United States). A steady increase has been reported over the last few decades in South America, Africa, and Asia (1). The pathogenesis of UC involves a complex interaction among the host genetic background, microbial shifts, and environmental cues, leading to inappropriate and chronic activation of the mucosal immune system (2, 3). Despite efforts for so many decades, the complex pathogenesis of UC has not yet been fully understood.

A number of population studies in various parts of the world have shown that patients with UC with longer disease duration (≥10 years) have a higher risk of developing ulcerative colitis-associated carcinogenesis (UCAC) when compared to those with shorter duration. Cumulative risk of colorectal cancer (CRC) for patients with UC was found to be 2% at 10 years, 8% at 20 years, and 18% at 30 years (4). Various transcriptomic studies have been carried out in the UC field, including discrimination between disease subtypes (Crohn’s disease vs. UC), disease phase (active/mildly active/inactive), colon biopsy locations, genders, etc. (5–7). However, the effects of different disease durations on transcriptome have not yet been explored systematically. Colonoscopy detection of neoplasia has long been employed as the standard procedure, and biomarkers could be a complement to colonoscopy surveillance for detecting UCAC at an early stage (8). Therefore, our study aimed to identify transcriptomic changes in patients with UC of different disease durations developing into UCAC and to provide a foundation study in the development of molecular biomarkers.

## Materials and Methods

**Figure 1:**
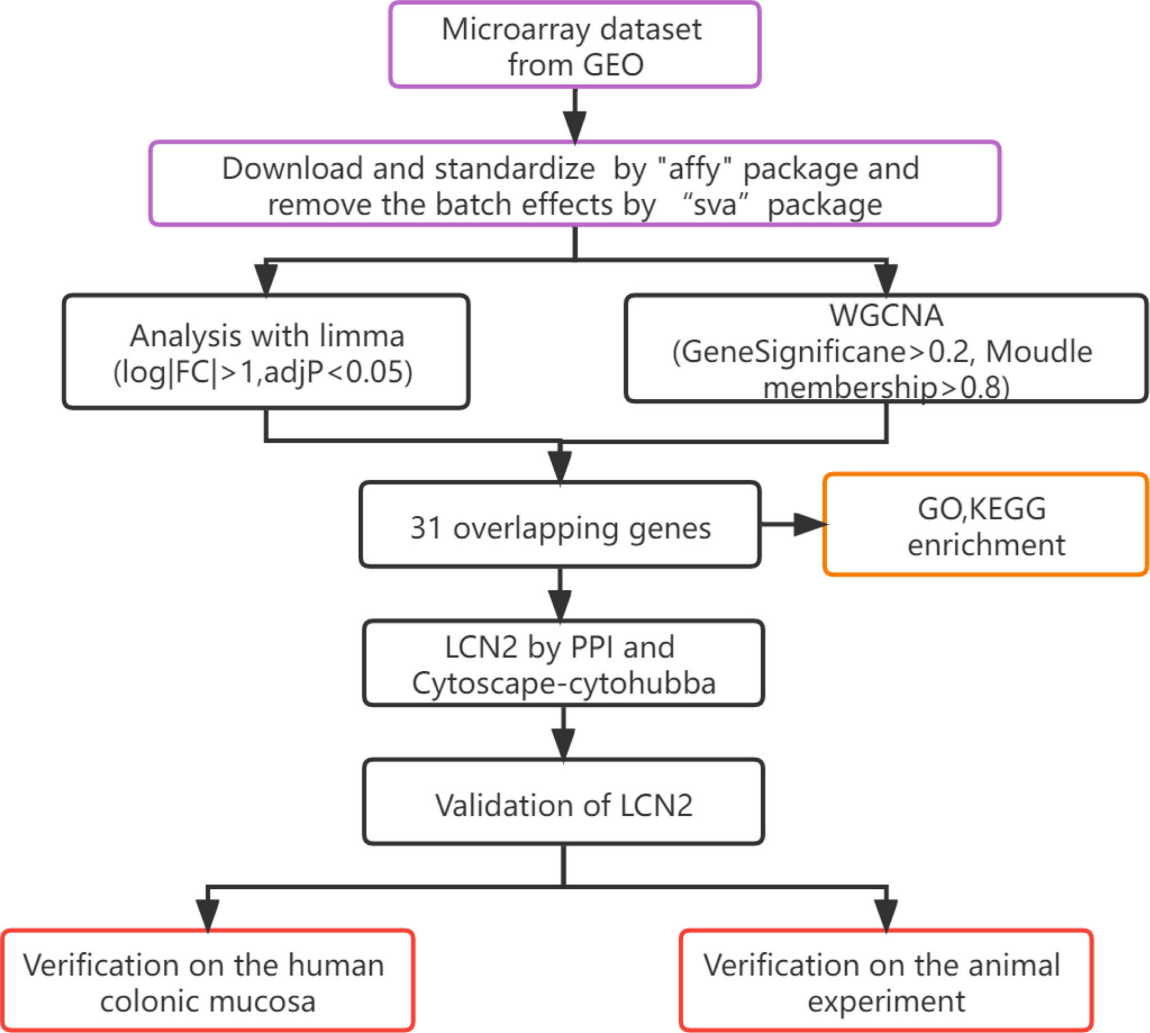
Flow diagram of the analysis process.

The workflow of hub gene extraction and creation pipeline were shown in **Figure 1**.

### Datasets From Gene Expression Omnibus Database

The gene expression datasets were obtained through the Gene Expression Omnibus (GEO) database (http://www.ncbi.nlm.nih.gov/geo). The microarray studies were searched using the following terms: “Ulcerative Colitis”, “Inflammatory Bowel Disease”, “*Homo sapiens*”, “gene expression” and “microarray”. Statistical group comparisons of UC were divided into two parts, including long-duration UC (≥10 years) and short-duration UC (<10 years). The datasets were included according to the following eligibility criteria: 1) with at least 10 total samples; 2) includes at least five cases and five controls; 3) the age range of patients undergoing biopsy should be from 18 to 70 years; 4) should contain information with regard to age and disease duration; 5) the raw data or gene expression profiling carried out by array should be available in the GEO; and 6) the samples of baseline data should not receive biologics or any steroid medication.

### Data Preprocessing of Gene Expression Omnibus Datasets

The gene expression matrix and related annotation document for each array dataset were downloaded from the GEO database. After we screened out the dataset that can be used for subsequent analysis, we used the “affy” package to download and standardize the raw “CEL” files of the microarray data (9). RNA sequencing data were transformed by using the “voom” algorithm in order to convert count data to values similar to those resulting from microarrays (10). We used the corresponding annotation document to map the microarray probes to ENTREZ ID. The 4 datasets (5, 11–13) (GSE114603, GSE38713, GSE47908, and GSE37283) were integrated to significantly increase the sample sizes, so as to avoid the single dataset leading to unreliable results and reducing the validity of the bioinformatics analysis. Heterogeneity and potential variables are generally recognized as the main sources of bias and variability in high-throughput experiments. The merged data were preprocessed by the sva package to remove the batch effect and heterogeneity among various datasets (14). If multiple probes were mapped to the same symbol, then the mean value was adopted.

### Identification of the Differentially Expressed Genes

The “LIMMA” (Linear Model of Chip Data) R software package was used to determine the microarrays. The microarrays were then divided into short-duration UC, long-duration UC, UCAC, and the normal control group in the GEO. The |log2fold change (FC)| >1 and adjusted *P* < 0.05 were regarded as the cutoff criteria to determine the differentially expressed genes (DEGs).

### Identification of Co-Expression Modules Based on Weighted Gene Co-Expression Network Analysis

The co-expression networks facilitate network-based gene screening methods that can be used to identify the candidate biomarkers and therapeutic targets. In our research, the gene expression data map of the UC datasets was constructed, and the gene expression network of R (15) was constructed based on the weighted gene co-expression network analysis (WGCNA) package. Hierarchical clustering was performed on the research samples by distance and average methods to detect the outliers and remove the four abnormal samples. WGCNA was used to explore the modules of highly correlated genes among the samples of related modules to that of external sample traits. To build a scale-free network, soft powers of β = 6 were selected by using the function pickSoft Threshold. Next, the adjacency matrix was established by the following formula: aij = |sij|β (aij: adjacency matrix between gene i and gene j,sij: similarity matrix composed of Pearson correlations of all gene pairs, β: soft power Value) and was converted to a topological overlap matrix (TOM) and the corresponding degree of dissimilarity (1-TOM). After that, a hierarchical clustering dendrogram of 1-TOM matrix was constructed to divide similar gene expressions into different gene co-expression modules. To further identify the functional modules in a co-expression network, the module–trait associations and clinical trait information were calculated according to previous research studies (16, 17). Therefore, modules with high correlation coefficients were considered as candidates related to clinical features and selected for subsequent analyses.

### Construction of Protein–Protein Interaction and Screening of Hub Genes

In this study, the DEGs of UC datasets and the hub genes of the selected WGCNA modules were intersected to obtain the overlapping genes. The online tool STRING (18) is designed for predicting protein–protein interactions (PPIs) and to construct a PPI network model by selecting genes with a score of ≥0.4. To explore the Gene Ontology (GO) of selected genes, the Metascape database (http://metascape.org) was used to study the functions among the genes of interest, with a cutoff criterion of *P* < 0.05 (19). GO annotation contains three sub-ontologies, biological process (BP), cell component (CC), and molecular function (MF), and these can identify the biological properties of genes and genomes of all organisms (20). The Kyoto Encyclopedia of Genes and Genomes (KEGG) Database was applied for pathway enrichment analysis, and significant (*P* < 0.05) genes were selected.

### Elucidation of Hub Gene Expression Pattern

To get the hub genes with the highest risk of carcinogenesis related to disease duration, we confirmed the expression patterns of the overlapping genes in short-duration UC, long-duration UC, UCAC, the normal control group, and selected hubs by one-way ANOVA (* *P* < 0.05; ** *P* < 0.01; *** *P* < 0.001). In GEO94648 (21) blood samples and GEO11223 (22) colorectal samples, the expression pattern of the hub gene in the short-duration UC, long-duration UC, and normal tissue was repeatedly verified to observe its expression characteristics. Limited by the current database, we did not find more datasets containing tissue of UCAC in the blood and colorectal samples.

### Verification on the Animal Experiment

#### Animals

All experimental procedures that involved mice in this study were approved by the Animal Ethics Committee of Beijing University of Chinese Medicine (No. BUCM-4-2020122103-4168) in strict accordance with the Guide for the Care and Use of Laboratory Animals published by the Ministry of Science and Technology of China. Male C57BL/6 mice (weight 20 ± 2 g) were purchased from Weitonglihua Laboratory Animal Research Center at Beijing (Number: SCXK-2019-0013). During the adaptation period of 7 days before modeling, mice were housed under standard conditions of temperature and humidity with freedom of access to water and food and were subjected to a 12-h light/dark cycle.

#### Modeling

Colitis-associated cancer (CAC): Mice received a single intraperitoneal (i.p.) injection of azoxymethane (AOM) (10 mg/kg). One week later, they were exposed to 2% dextran sulfate sodium salt (DSS) (wt/vol) in drinking water for 7 consecutive days followed by 14 days of regular drinking water. This cycle was then repeated twice. Chronic colitis: Two weeks later, they were exposed to 2% DSS (wt/vol) in drinking water for 7 consecutive days followed by 14 days of regular drinking water. This cycle was then repeated twice. Acute colitis: Nine weeks later, they were exposed to 2% DSS (wt/vol) in drinking water for 7 consecutive days (23).

DSS was purchased from MP Biomedicals (Santa Ana, CA, USA). AOM was purchased from Sigma Aldrich (St. Louis, MO, USA). Body weight was assessed once a week throughout the duration of the experiment, and stool of the four groups was kept in the last 3 days for follow-up testing. All mice were sacrificed by cervical dislocation at the beginning of the 11th week. The colons were excised from the ileocecal junction to anus, and the length and weight of colons were measured, after which the colons were examined macroscopically and tumor numbers were counted.

#### Histopathological Analysis

Formalin-preserved colons were dehydrated and embedded in paraffin by standard techniques. Subsequently, paraffin-embedded samples were sectioned at 5 µm and stained with hematoxylin and eosin (H&E).

#### Immunofluorescence Staining

The protein expression levels of lipocalin-2 (LCN2) in the normal, acute colitis, chronic colitis, and CAC mice were detected by immunofluorescence staining. The sections were then incubated with an anti-LCN2 antibody (A2092, ABclonal, Cambridge, MA; 1:50 dilution) at 4°C overnight and then washed with phosphate buffered solution (PBS) three times. The sections were then co-incubated with secondary antibody (GB25303, Servicebio, Wuhan, China; 1:400 dilution). The nuclei were stained with 4',6-diamidino-2-phenylindole (DAPI) (Invitrogen). The images were taken with confocal microscope (CSIM100, SUNNY, Beijing, China).

#### Enzyme-Linked Immunosorbent Assay

The extracted colonic tissues were weighed and homogenized with tissue extraction reagent on ice for 3 min using a homogenizer. Interleukin (IL)-6, tumor necrosis factor (TNF)-α, myeloperoxidase (MPO), calprotectin (CALP), and LCN2 in the colonic mucosa and CALP and LCN2 in the stool were measured using mouse ELISA kits (Cusabio Biotech, Newark, DE, USA). Four samples of each group were randomly chosen for analysis.

#### Real-Time PCR and Western Blot

The mRNA expression levels of LCN2 in the normal, acute colitis, chronic colitis, and CAC mice were detected by RT-PCR. The LCN2 primer used for the amplification was as follows: 5′- CACCACGGACTACAACCA-3′ and 5′-ACACTCACCACCCATTCA-3′ (Sangon Biotech, China). Here, β-actin was regarded as the internal control. The reliability of the PCR results was verified in correspondence with the dissolution curve. The cycle threshold (Ct, which is the inflection point on the amplification power curve) was calculated, and the relative gene expression was calculated using the 2^-ΔΔCt^ method [ΔCt = Ct (target gene) - Ct β-actin, ΔΔCt = ΔCt (target gene) - ΔCt (β-actin)]. Five samples of each group were randomly chosen for RT-PCR. The protein expression levels of LCN2 in the normal, acute colitis, chronic colitis, and CAC mice were detected by Western blot. The Anti-LCN2 antibody (ab216462, 1:1,000) and the Anti-GAPDH antibody (ab9485, 1:2,500) were purchased from Abcam. The target protein bands were developed by using enhanced chemiluminescence Western blotting detection reagent, and densities were quantified using Total Lab Quant V11.5 (Newcastle upon Tyne, UK). Three samples of each group were randomly chosen for Western blotting analysis.

### Verification on the Human Colonic Mucosa

#### Immunofluorescence Staining

The intestinal mucosal pathology reports of patients who have undergone colonoscopy in the Department of Pathology, Dongfang Hospital, Beijing University of Chinese Medicine, including 6 cases of long-duration UC, 6 cases of short-duration UC, 6 cases of normal, and 2 cases of UCAC were obtained. As previous description, the protein expression of LCN2 was then detected by immunofluorescence method.

#### Human Protein Atlas Analyses

The Human Protein Atlas (HPA) database (https://www.proteinatlas.org) was used for identifying the expression level of LCN2 protein in CRC and normal through immunohistochemistry (IHC) staining. In addition, IHC images were downloaded from HPA, which is an online tool that contains IHC test results, showing the distribution and expression of proteins in various human normal and tumor tissues (24).

### Statistical Analysis

Statistical analyses were performed by RStudio software and the R software (version 3.6.1), SPSS version 25.0 (SPSS Inc., USA), and GraphPad Prism 8.0 (GraphPad Inc., USA). Mean ± standard deviation (SD) was used to describe the continuous variables of normal distribution. Median and quartile were used to describe the continuous variable of skewed distribution. One-way analysis of variance (ANOVA) was applied to analyze the data. The least significant difference (LSD) test was performed under the assumption of equal variances; otherwise, Dunnett’s T3 test was applied for data with unequal variances. Statistical significance of the difference was set at *P* < 0.05.

## Results

### Dataset and Preprocessing

The study finally screened out six datasets, including GSE114603, GSE38713, GSE47908, GSE37283, GSE94648, and GSE11223, and among these, the clinical information of GSE47908 and GSE114603 was provided by the author. The information of each dataset was extracted separately (**Table 1**). After preprocessing the UC datasets, the data and the eliminating outliers, a total of 17,526 genes were identified as the gene list. In addition, hierarchical cluster analysis (R: hclust function) was used to eliminate outliers and reduced the samples to 39 normal, 40 short-duration UC, 26 long-duration UC, and 15 UCAC samples.

**Table 1:** Characteristics of studies composing the dataset gene expression.

Dataset	Healthy controls	Short-duration UC (<10 years)	Long-duration UC (≥10 years)	UC neoplastic/cancer	Tissues	Platform	Country	Year
GSE38713	13	10	10	NA	Colorectal	GPL570	Spain	2012
GSE114603	6	9	4	NA	Colorectal	GPL14951	Spain	2018
GSE47908	15	21	12	5	Colorectal	GPL570	Denmark	2014
GSE37283	5	NA	NA	10	Colorectal	GPL13158	USA	2012
GSE94648	14	8	14	NA	Blood	GPL19109	Spain	2017
GSE11223	63	28	25	NA	Colorectal	GPL1708	USA	2008

UC, ulcerative colitis; NA, not available.

### Construction of Weighted Gene Co-Expression Modules

**Figure 2:**
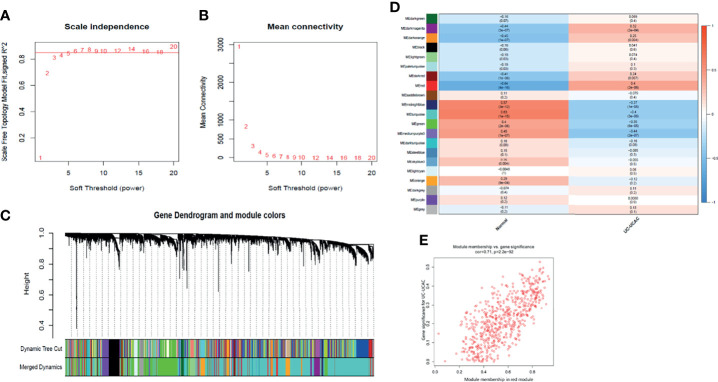
Identification of modules associated with the clinical information in the WGCNA. **(A)** Analysis of the scale‐free fit index for various soft‐thresholding powers (β). **(B)** Analysis of the mean connectivity for various soft‐thresholding powers. **(C)** Dendrogram of all differentially expressed genes clustered based on a dissimilarity measure (1‐TOM). **(D)** Module–trait relationships. Each cell contains the corresponding correlation and *P* value. **(E)** A scatter plot of gene significance for UC-UCAC vs. the membership in the red module. TOM, topological overlap matrix; WGCNA, weighted gene co-expression network analysis.

To find the functional clusters related to the carcinogenesis of UC patients, the WGCNA software package was used to construct a gene co-expression network from the processed UC datasets. Before constructing the weighted co-expression network, an appropriate soft threshold β was chosen. After calculation, the soft threshold β = 6 was chosen to construct the gene module when the correlation coefficient was close to 0.85 (**Figures 2A, B**). Following the determination of the soft threshold, the correlation matrix and adjacency matrix of the gene expression profile of UC samples were calculated and subsequently converted into TOM in the light of the WGCNA algorithm. The standard for merging similar modules was to set MEDissThres to 0.3, and 21 modules were generated and assigned the unique color (**Figure 2C**, excluding gray modules that are not assigned to any cluster). After that, the heat map of the module–trait relationship was drawn to assess the association between each module and the clinical characteristics (normal and UC-UCAC). Among the clinical traits, the trait of UC-UCAC group included different traits such as normal, short duration of UC, long duration of UC, and UCAC, while the trait of the normal group was divided into normal and patients. The results showed that the red module had the highest correlation with the evolution of UC to UCAC (r = 0.4, *P* = 2e-06) (**Figure 2D**). Subsequently, an intramodular analysis of gene significance (GS) and module membership (MM) of the genes in the red module was performed. A high correlation coefficient of GS and MM was found in the red module (cor = 0.71, *P* = 2.2e-92) (**Figure 2E**). The hub genes in the module were screened out according to gene significance of >0.2 and module membership of >0.8, among which 96 genes are obtained from the red module.

### Identification of Overlapping Genes Between UC Datasets and Co-Expression Module

**Figure 3:**
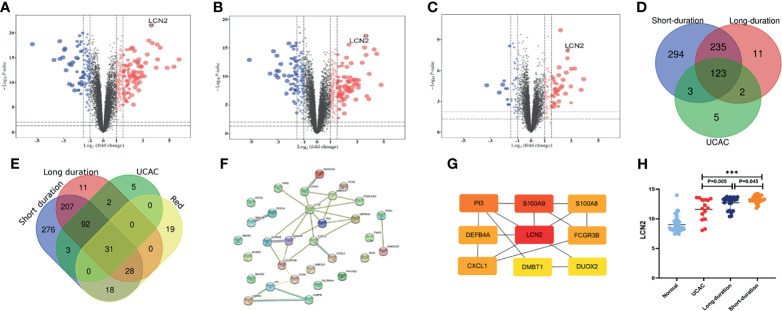
Identification of differentially expressed genes (DEGs) among Gene Expression Omnibus (GEO) datasets. **(A)** Short-duration ulcerative colitis (UC) vs. normal. **(B)** Long-duration UC vs. normal. **(C)** Ulcerative colitis-associated carcinogenesis (UCAC) vs. normal. **(D)** Overlapping genes in UC datasets. **(E)** Overlapping genes in UC datasets, hub genes in red module. **(F)** Protein–protein interaction (PPI) network of 31 overlapping genes. **(G)** The top nine key genes by the maximal clique centrality (MCC). **(H)** Expression levels of LCN2 in UC datasets. ***P < 0.001.

The genes with the cutoff criteria of |logFC| ≥1 and adjusted *P* value of <0.05 were regarded as DEGs. Compared with normal tissues, 655 DEGs in short-duration UC (**Figure 3A**), 371 DEGs in long-duration UC (**Figure 3B**), and 133 DEGs in UCAC (**Figure 3C**) were found by using the LIMMA software package. There were 123 overlapping genes among the three parts (**Figure 3D**). Subsequently, the intersection of the overlapping genes in UC datasets and hub genes in red module was obtained. A total of 31 overlapping genes were extracted for verification (**Figure 3E**, **Supplementary Table S1**).

### Protein–Protein Interaction Network and Functional Enrichment Analysis

**Figure 4:**
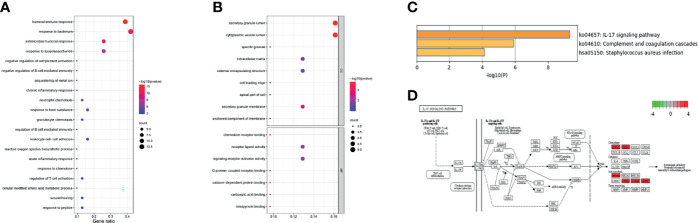
Gene Ontology (GO) and Kyoto Encyclopedia of Genes and Genomes (KEGG) enrichment analysis. **(A)** The biological processes (BP). **(B)** The molecular function (MF) and cell composition (CC). **(C)** The KEGG pathway enrichment. **(D)** Data on KEGG graph rendered by Pathview.

To further explore the potential functions of the 31 overlapping genes, STRING database was applied to build a PPI network of overlapping genes, with a total of 31 nodes and 29 edges (**Figure 3F**). The CytoHubba plugin MCC algorithm was used to select the top nine key genes (LCN2, S100A9, P13, S100A8, FCGR3B, CXCL1, DEFB4A, DUOX2, DMBT1) from the PPI network (**Figure 3G**), and LCN2 ranked first in 9 of 12 methods in cytoHubba plugin (**Supplementary Table S2**). The expression levels of overlapping genes in different disease durations of UC, UCAC, and normal tissues were proven, and LCN2 was obtained (*P* < 0.01) among the nine key genes with the highest risk of carcinogenesis (**Figure 3H**). Subsequently, the Metascape database was used for gene enrichment analysis of 31 overlapping genes. After screening out by GO enrichment analysis, several groups of enriched genes were presented in **Figure 4**. The biological processes (BP) of the 31 genes were mainly from humoral immune response, response to bacterium, negative regulation of complement activation, and sequestering of metal ion, as well as response to toxic substance and reactive oxygen species biosynthetic process (**Figure 4A**, **Supplementary Table S3**). The molecular function (MF) was mainly enriched in receptor ligand activity and signaling receptor activator activity, and the cellular composition (CC) was mainly involved in specific granule lumen, secretory granule lumen, and cytoplasmic vesicle lumen (**Figure 4B**, **Supplementary Table S4**). KEGG pathway analysis indicated that the genes might influence the occurrence and progression of UCAC by participating in pathways such as IL-17 signaling pathway and complement and coagulation cascades (**Figure 4C**), and six genes were enriched in IL-17 signaling pathway (**Figure 4D**).

### Expression Verification of LCN2 in Animals

**Figure 5:**
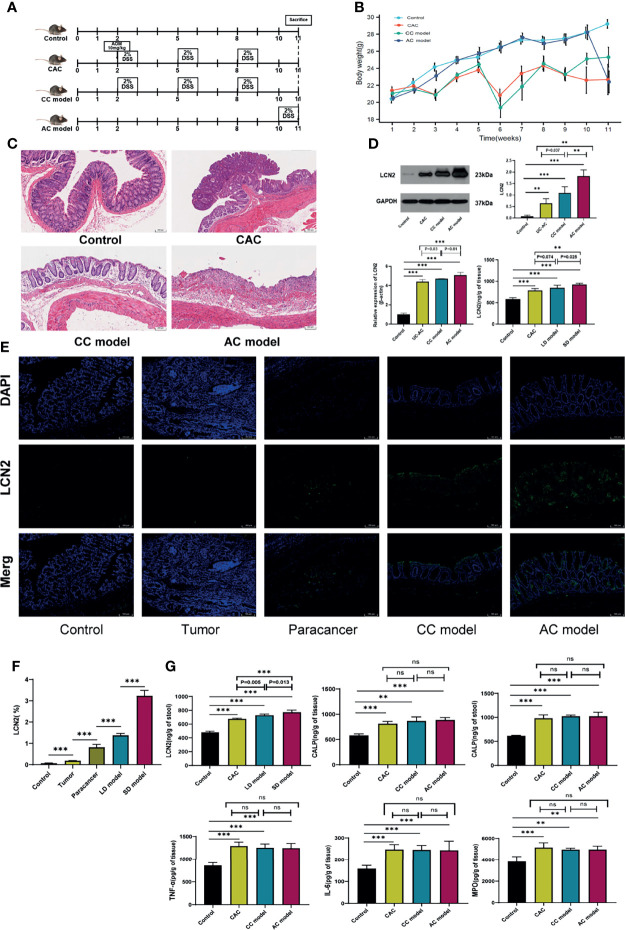
Protein expression verification in animals. **(A)** Schematic overview of control, acute colitis (AC), chronic colitis (CC) model, and colitis-associated cancer (CAC) model. **(B)** Weight loss in mouse of control and three modeling groups. **(C)** H&E staining of colon sections of mouse at week 11. **(D)** Lipocalin-2 (LCN2) expression level in colorectal mucosa was detected by RT-PCR, Western blot, and ELISA. **(E)** Immunofluorescence of LCN2 in control, tumor, paracancer, long-duration (LD) ulcerative colitis (UC) model, and short-duration (SD) UC model. **(F)** Semiquantitative analysis of immunofluorescence images of LCN2. **(G)** Calprotectin (CALP), myeloperoxidase (MPO), interleukin (IL)-6, and tumor necrosis factor (TNF)-α expression levels in colorectal mucosa and LCN2 in stool were detected by ELISA. **P < 0.01, ***P < 0.001; ns, not significant.

To explore the role of LCN2 in the duration of UC disease, the models (acute colitis, chronic colitis, CAC) were built (**Figure 5A**). As shown in **Figure 5B**, the significant body weight loss was observed in three modeling groups when compared with the control group. Histologic evaluation showed colon crypts and mucosal damage in acute colitis, crypt atrophy and abnormal epithelial in chronic colitis, and mucosal structure disorder in CAC (**Figure 5C**). As demonstrated in RT-PCR and Western blot, the expression levels of LCN2 in acute colitis, chronic colitis, and CAC were all higher than those of normal (*P* < 0.001), acute colitis was higher than that of the chronic colitis (*P* ≤ 0.01), and chronic colitis was higher than that of CAC (*P* < 0.05) (**Figure 5D**). Meanwhile, immunofluorescence staining confirmed a consistent expression trend (**Figures 5E, F**). Levels of LCN2 and CALP in colonic mucosa and stool were markedly elevated in AOM/DSS- and DSS-induced mice relative to that in the control group (*P* < 0.001). Moreover, we observed that LCN2 expression of chronic colitis could dramatically decrease compared with that of acute colitis (*P* < 0.05). Indeed, we did not make a significant difference of LCN2 expression (*P* = 0.074) between chronic colitis and CAC in the tissue of animal model. However, a downward trend could still be observed between chronic colitis and CAC. To a large extent, chronic colitis and CAC model are consistent in the same modeling time span, it is more likely to result in non-significant differences of LCN2 expression. There was no difference in the expression of IL6, TNF-α, MPO, and CALP in the disease course progresses (**Figure 5G**).

### Protein Expression Verification of LCN2 in Human

**Figure 6:**
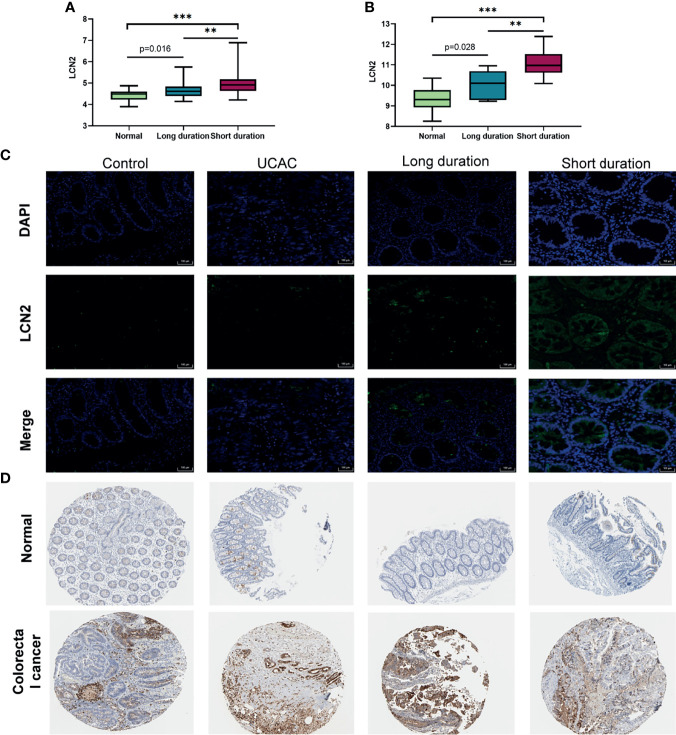
Protein expression verification in human. **(A)** Expression levels of lipocalin-2 (LCN2) in colorectal mucosa of GSE11223. **(B)** Expression levels of LCN2 in blood sample of GSE94648. **(C)** Immunofluorescence of LCN2 in normal, short-duration ulcerative colitis (UC), long-duration UC, and ulcerative colitis-associated carcinogenesis (UCAC). **(D)** LCN2 in normal colon tissues and colorectal cancer (CRC) from the Human Protein Atlas (HPA) database. **P < 0.01, ***P < 0.001.

The expression levels of LCN2 among the long-duration UC, short-duration UC, and normal tissues in colorectal samples (GSE11223) and blood samples (GEO94648) were further investigated and shown in **Figures 6A, B**. The LCN2 immunofluorescence of intestinal mucosa revealed that the LCN2 of short-duration UC was significantly higher than that of the long-duration UC, that of the long-duration UC was higher than that of the UCAC, and those of the short-duration UC and long-duration UC were both higher than that of the normal (**Figure 6C**). However, there was no difference of LCN2 expression level between UCAC and normal tissues. The cause might be largely explained by the inclusion of a small sample size. At the same time, based on the HPA database, the protein levels of LCN2 gene in tumor tissues were found to be significantly higher than that in normal tissues (**Figure 6D**).

## Discussion

LCN2, also referred to as neutrophil gelatinase–associated lipocalin (NGAL) or siderocalin, is a peptide produced by macrophages, neutrophil granulocytes, and other immune and parenchymal cells. It has an antimicrobial function by binding catechol-type siderophores with an extremely high affinity (25). Moreover, it is linked to several physiological roles, including transporting hydrophobic ligands across cell membranes, modulating immune responses, maintaining iron homeostasis, and promoting epithelial cell differentiation (25–28). So, LCN2 is a chemically highly stable protein that makes it as an interesting biomarker candidate (29). Stallhofer et al. (30) have confirmed the excessive *de novo* synthesis of LCN2 is localized in human colonic epithelial cells, and serum LCN2 has the potential to be a biomarker of active UC. Fecal LCN2 could be used as a biomarker for detecting endoscopic activity in patients with inflammatory bowel diseases. In deep remission, expressions of LCN2 but not CALP remained elevated in the rectum mucosa of UC patients (31). While our study was performed, some reports on the role of LCN2 in other colitis were published except UC, including collagenous colitis and necrotizing enterocolitis. LCN2 was also identified as a potential fecal biomarker (32, 33). Previous review displays that expression of LCN2 in CAC is variable (34). Some *in vitro* and *in vivo* studies in the literature support the role of LCN2 as a tumor promoter (35), but the difference is that in most of the current studies, LCN2 is generally considered to be a protective factor in CRC (36–38). LCN2 expression was significantly lower in metastatic or advanced-stage CRC than that in non-metastatic or early-stage CRC (39). Therefore, there is a clear and urgent need to confirm changes of LCN2 expression in the process of carcinogenesis from UC, so as to develop diagnostic tools to better monitor the progression of disease.

UC patients with long disease duration have a higher risk of developing CAC compared with patients with short-duration UC. LCN2 has already been proven to be a major downregulated differential gene associated with the duration of UC disease by Low et al. (8), and this was consistent with our study results. On the basis of that, our study compared samples of healthy people vs. samples from patients with the disease in databases, including short-duration UC, long-duration UC, and UCAC. The expression level of LCN2 was significantly higher in the diseased tissues than in the normal tissues. Noteworthy, the expression of LCN2 is significantly decreased in UCAC when compared to long-duration UC, which correlates to the disease duration of UC. We identify that LCN2 is associated with the risk of carcinogenesis based on disease duration among the hub genes. Collectively, from the perspective of BP, CC, and KEGG analyses, LCN2 showed close relation to antibacterial response and immune activation. Based on the previously reported involvement of LCN2 in chemokine induction and in the recruitment of neutrophils at the sites of infection or tissue injury (40). LCN2 could enhance Th17 type responses and cell recruitment (41).

Given the nature of our study, the average ages of healthy people and patients with short-duration UC, long-duration UC, and UCAC were 43 ± 13 years, 36 ± 13 years, 47 ± 11 years, and 50 ± 8 years, respectively. Difference in age might be inevitable. Li et al. (42) have suggested that the molecular pathways in diseased tissue samples are similar between adult and pediatric UC patients. However, there is yet to prove the direct effects of age on transcriptomic studies. Another point to be noted is that verification of proteomics sample obtained in the study was from a single center. A larger population study group is warranted to make any definitive conclusions in this matter. Additionally, a stronger effect of anti-TNF drugs induced a decrease in LCN2 (43), and therefore, the patients who have received treatment were excluded in the study, and only the baseline state was used.

Long-duration UC acts as a risk factor for the development of UCAC, and the transcriptomic at different disease duration intervals can aid in determining the progress, along with colonoscopy. The expression levels of LCN2 in disease groups are increased when compared with the normal group, but the expression levels of LCN2 are gradually reduced in tissues of short-duration UC, long-duration UC, and UCAC. The condition also applies to the serum LCN2, and the expression trend of LCN2 in feces shows consistent results with that of blood and tissue. Because blood and feces are easier to detect than tissues, LCN2 expression is more convenient for disease surveillance. In addition, for the monitoring of colon cancer related with course, fecal LCN2 has more advantages than fecal CALP in our experimental verification. However, there are a few limitations in this study. Firstly, the results are only based on the current database of known gene sequences and transcripts. Because the experiments are done in different centers, this kind of missing information are inevitable in public databases. No research on children with regard to disease duration and prognosis exists. Then, the chronic UC is similar to that of cancer modeling in the animal experiment; it should be further verified in clinical samples. Finally, the trend of LCN2 expression in disease tissues is clear basically, but corresponding threshold of LCN2 in blood and feces is a significant issue worthy of continued investigation. Noteworthy, other carcinomas, neuropathy, and metabolic diseases (44–46) have a greater impact on the level of LCN2 in the blood, and the detection of stool may be more beneficial for diagnosis value of UCAC in subsequent use.

## Conclusions

In conclusion, we analyzed transcriptomic changes in patients with UC of different disease durations developing into UCAC to further identify the promising diagnostic biomarkers. We showed that the expression level of LCN2 was significantly negatively correlated with the risk of UCAC. The expression level of LCN2 in short-duration UC was higher than that of long-duration UC, that of long-duration UC was higher than that of UCAC, and those of UC and UCAC were all higher than that of the normal. We then validated this trend in human proteomics and animal experiment. In addition, we also discovered that the expression trend of LCN2 in blood and stool samples was consistent with that in colorectal mucosa. However, LCN2 expression in blood sample was easily affected by a multitude of factors. Stool detection may have more advantages for diagnosis value of early-stage UCAC as a complement to colonoscopy surveillance.

## Data Availability Statement

The datasets presented in this study can be found in online repositories. The names of the repository/repositories and accession number(s) can be found in the article/**Supplementary Material**.

## Ethics Statement

Ethical review and approval were not required for the study on human participants in accordance with the local legislation and institutional requirements. Written informed consent for participation was not required for this study in accordance with the national legislation and the institutional requirements. The animal study was reviewed and approved by the Animal Ethics Committee of Beijing University of Chinese Medicine.

## Author Contributions

FSK and YC contributed equally to the study as first authors. YC and FSK: study concept and design. FSK and JJL: acquisition of data. FSK and LS: analysis of data. YC: article writing. YC, JJL, YYL, RS, GYP, and JXL: interpretation of data and critical revision of the article. JXL consulted fees or honoraria from. All authors contributed to the article and approved the submitted version.

## Funding

This work was supported by the National Key R&D Program of China [2018YFC1705403, 2018YFC1705405] and Innovation “One Hundred Million” Talent Project Qihuang Scholar.

## Conflict of Interest

The authors declare that the research was conducted in the absence of any commercial or financial relationships that could be construed as a potential conflict of interest.

## Publisher’s Note

All claims expressed in this article are solely those of the authors and do not necessarily represent those of their affiliated organizations, or those of the publisher, the editors and the reviewers. Any product that may be evaluated in this article, or claim that may be made by its manufacturer, is not guaranteed or endorsed by the publisher.
