# Supplementary material for: LCN2 as a Potential Diagnostic Biomarker for Ulcerative Colitis-Associated Carcinogenesis Related to Disease Duration
Source: Front Oncol. 2022 Jan 17;11:793760. doi: 10.3389/fonc.2021.793760 (PMC8801604; doi:10.3389/fonc.2021.793760)
Supplement: Supplementary file 1 [file Table_1.docx]

Supplementary Material

**Supplementary Table 1. 31 overlapping genes.**

| Gene ID | Symbol | Description | Protein Function (Protein Atlas) | Subcellular Location (Protein Atlas) |
| --- | --- | --- | --- | --- |
| 79931 | TNIP3 | TNFAIP3 interacting protein 3 | Predicted intracellular proteins |  |
| 10158 | PDZK1IP1 | PDZK1 interacting protein 1 |  | Cytosol (Approved); Additional: Nuclear speckles |
| 2921 | CXCL3 | C-X-C motif chemokine ligand 3 | Predicted secreted proteins |  |
| 3620 | IDO1 | indoleamine 2,3-dioxygenase 1 | ENZYME proteins:Oxidoreductases; Predicted intracellular proteins; Enzymes; Cancer-related genes:Candidate cancer biomarkers | Cytosol (Supported); Additional: Mitochondria;Nucleoplasm;Vesicles |
| 3158 | HMGCS2 | 3-hydroxy-3-methylglutaryl-CoA synthase 2 | Disease related genes; ENZYME proteins:Transferases; Predicted intracellular proteins; Potential drug targets; Enzymes | Mitochondria (Supported) |
| 6947 | TCN1 | transcobalamin 1 | Predicted secreted proteins |  |
| 1604 | CD55 | CD55 molecule (Cromer blood group) | Blood group antigen proteins; CD markers; Disease related genes; Predicted intracellular proteins; Predicted secreted proteins |  |
| 3426 | CFI | complement factor I | Disease related genes; Peptidases:Serine-type peptidases; Potential drug targets; ENZYME proteins:Hydrolases; Predicted secreted proteins; Enzymes |  |
| 1755 | DMBT1 | deleted in malignant brain tumors 1 | Disease related genes; Predicted secreted proteins | Centriolar satellite (Approved) |
| 405753 | DUOXA2 | dual oxidase maturation factor 2 | Disease related genes |  |
| 8876 | VNN1 | vanin 1 | Predicted intracellular proteins; Enzymes; ENZYME proteins:Hydrolases |  |
| 50506 | DUOX2 | dual oxidase 2 | Disease related genes; ENZYME proteins:Oxidoreductases; Transporters:Transport Electron Carriers; Potential drug targets; Enzymes |  |
| 2215 | FCGR3B | Fc fragment of IgG receptor IIIb | CD markers; Predicted secreted proteins; FDA approved drug targets:Biotech drugs; Predicted intracellular proteins |  |
| 25825 | BACE2 | beta-secretase 2 | Predicted intracellular proteins; Transporters:Accessory Factors Involved in Transport; Peptidases:Aspartic-type peptidases; ENZYME proteins:Hydrolases; Enzymes |  |
| 3669 | ISG20 | interferon stimulated exonuclease gene 20 | Predicted intracellular proteins; Enzymes; ENZYME proteins:Hydrolases |  |
| 2919 | CXCL1 | C-X-C motif chemokine ligand 1 | Predicted secreted proteins; Cancer-related genes:Candidate cancer biomarkers |  |
| 9246 | UBE2L6 | ubiquitin conjugating enzyme E2 L6 | Predicted intracellular proteins; Enzymes; ENZYME proteins:Transferases | Cytosol (Approved) |
| 5967 | REG1A | regenerating family member 1 alpha | Predicted secreted proteins |  |
| 5243 | ABCB1 | ATP binding cassette subfamily B member 1 | CD markers; Disease related genes; Predicted intracellular proteins; Transporters:Primary Active Transporters; Cancer-related genes:Mutational cancer driver genes; Potential drug targets; ENZYME proteins:Hydrolases; Cancer-related genes:Candidate cancer biomarkers; Enzymes | Nucleoplasm;Plasma membrane (Approved); Additional: Focal adhesion sites |
| 80221 | ACSF2 | acyl-CoA synthetase family member 2 | Predicted intracellular proteins | Cytosol;Microtubules;Nucleoplasm (Uncertain) |
| 1673 | DEFB4A | defensin beta 4A | Predicted secreted proteins; Transporters:Transporter channels and pores |  |
| 5968 | REG1B | regenerating family member 1 beta | Predicted intracellular proteins; Predicted secreted proteins |  |
| 5105 | PCK1 | phosphoenolpyruvate carboxykinase 1 | Disease related genes; ENZYME proteins:Lyases; Predicted intracellular proteins; Potential drug targets; Enzymes; Citric acid cycle related proteins |  |
| 725 | C4BPB | complement component 4 binding protein beta | Predicted secreted proteins |  |
| 5209 | PFKFB3 | 6-phosphofructo-2-kinase/fructose-2,6-biphosphatase 3 | Predicted intracellular proteins; Enzymes; ENZYME proteins:Hydrolases; ENZYME proteins:Transferases | Nucleoplasm (Enhanced) |
| 3934 | LCN2 | lipocalin 2 | Candidate cardiovascular disease genes; Predicted secreted proteins | Endoplasmic reticulum (Supported) |
| 722 | C4BPA | complement component 4 binding protein alpha | Predicted secreted proteins |  |
| 6279 | S100A8 | S100 calcium binding protein A8 | Predicted intracellular proteins; Predicted secreted proteins; Cancer-related genes:Candidate cancer biomarkers | Intermediate filaments (Supported); Additional: Cytosol |
| 5266 | PI3 | peptidase inhibitor 3 | Predicted secreted proteins | Plasma membrane (Approved) |
| 11254 | SLC6A14 | solute carrier family 6 member 14 | Potential drug targets; Disease related genes; Transporters:Electrochemical Potential-driven transporters | Vesicles (Approved) |
| 6280 | S100A9 | S100 calcium binding protein A9 | Predicted intracellular proteins; Predicted secreted proteins; Cancer-related genes:Candidate cancer biomarkers | Cell Junctions;Cytosol;Nucleoplasm (Supported) |

**Supplementary Table 2. Top 10 genes of 12 methods in CytoHubba pluggin.**

| **Rank** | **MCC** | **MNC** | **Degree** | **EPC** | **Bottleneck** | **Cleseness** | | **Radiality** | | **Betweenness** | **Stress** | **ClusteringCoefficient** | **DNNC** | **EcCentricity** |
| --- | --- | --- | --- | --- | --- | --- | --- | --- | --- | --- | --- | --- | --- | --- |
| **1** | LCN2 | LCN2 | LCN2 | LCN2 | LCN2 | LCN2 | LCN2 | | LCN2 | | LCN2 | C4BPA | S100A8 | S100A8 |
| **2** | S100A9 | S100A9 | S100A9 | S100A9 | S100A9 | S100A9 | S100A9 | | FCGR3B | | FCGR3B | C4BPB | DEFB4A | S100A9 |
| **3** | PI3 | S100A8 | FCGR3B | PI3 | CXCL1 | CXCL1 | CXCL1 | | CD55 | | CD55 | S100A8 | PI3 | FCGR3B |
| **4** | S100A8 | DEFB4A | CXCL1 | CXCL1 | FCGR3B | PI3 | S100A8 | | CXCL1 | | CXCL1 | DEFB4A | C4BPA | CXCL1 |
| **5** | FCGR3B | PI3 | PI3 | FCGR3B | CD55 | FCGR3B | FCGR3B | | CFI | | CFI | S100A9 | C4BPB | CD55 |
| **6** | CXCL1 | C4BPA | DUOX2 | DEFB4A | DUOX2 | S100A8 | DEFB4A | | S100A9 | | S100A9 | CFI | CFI | CXCL3 |
| **7** | DEFB4A | C4BPB | S100A8 | S100A8 | CFI | DEFB4A | PI3 | | DUOX2 | | DUOX2 | PI3 | FCGR3B | DEFB4A |
| **8** | DUOX2 | CFI | CFI | DMBT1 | PI3 | DUOX2 | DUOX2 | | S100A8 | | S100A8 | FCGR3B | CXCL1 | LCN2 |
| **9** | DMBT1 | FCGR3B | DEFB4A | DUOX2 | DUOXA2 | PDZK1IP1 | PDZK1IP1 | | PI3 | | PI3 | CXCL1 | S100A9 | PI3 |
| **10** | CFI | CXCL1 | DMBT1 | PDZK1IP1 | S100A8 | DMBT1 | CD55 | | DMBT1 | | PDZK1IP1 | LCN2 | LCN2 | DUOX2 |

**Supplementary Table 3. Top 20 of Biological Process (GO BP) enrichment for 31 genes.**

| **Term** | **Description** | **Symbols** | **pvalue** |
| --- | --- | --- | --- |
| GO:0006959 | humoral immune response | C4BPA,C4BPB,CD55,DEFB4A,DMBT1,CXCL1,CXCL3,CFI,PI3,REG1A,REG1B,S100A9 | 1.82E-16 |
| GO:0009617 | response to bacterium | C4BPA,C4BPB,CD55,DEFB4A,DMBT1,CXCL1,CXCL3,LCN2,PCK1,PI3,S100A8,S100A9,TNIP3 | 1.63E-13 |
| GO:0019730 | antimicrobial humoral response | DEFB4A,DMBT1,CXCL1,CXCL3,PI3,REG1A,REG1B,S100A9 | 4.34E-13 |
| GO:0032496 | response to lipopolysaccharide | CD55,CXCL1,CXCL3,LCN2,PCK1,S100A8,S100A9,TNIP3 | 1.59E-09 |
| GO:0045959 | negative regulation of complement activation | C4BPA,C4BPB,CD55,CFI,PCK1,DMBT1 | 5.44E-08 |
| GO:0002713 | negative regulation of B cell mediated immunity | C4BPA,C4BPB,CD55 | 5.41E-07 |
| GO:0051238 | sequestering of metal ion | LCN2,S100A8,S100A9,VNN1,DEFB4A,DMBT1,PI3,BACE2,CD55,PCK1,REG1A,IDO1,DUOXA2 | 5.41E-07 |
| GO:0002544 | chronic inflammatory response | S100A8,S100A9,VNN1 | 7.88E-07 |
| GO:0030593 | neutrophil chemotaxis | CXCL1,CXCL3,S100A8,S100A9 | 3.72E-06 |
| GO:0009636 | response to toxic substance | LCN2,ABCB1,S100A9,SLC6A14,DUOX2 | 6.64E-06 |
| GO:0071621 | granulocyte chemotaxis | CXCL1,CXCL3,S100A8,S100A9 | 8.03E-06 |
| GO:0002713 | regulation of B cell mediated immunity | C4BPA,C4BPB,CD55 | 3.21E-05 |
| GO:0007159 | leukocyte cell-cell adhesion | CD55,PCK1,S100A8,S100A9,VNN1 | 3.53E-05 |
| GO:1903409 | reactive oxygen species biosynthetic process | LCN2,DUOX2,DUOXA2,VNN1,BACE2,HMGCS2,PCK1,REG1A,ABCB1 | 3.89E-05 |
| GO:0002526 | acute inflammatory response | LCN2,S100A8,VNN1 | 0.000206 |
| GO:1990868 | response to chemokine | CXCL1,CXCL3,REG1A | 0.000135 |
| GO:0050863 | regulation of T cell activation | CD55,IDO1,PCK1,VNN1 | 0.000347 |
| GO:0006575 | cellular modified amino acid metabolic process | VNN1,DUOX2,DUOXA2 | 0.000949 |
| GO:0042060 | wound healing | C4BPB,REG1A,S100A8,DUOX2 | 0.000888 |
| GO:1901652 | response to peptide | LCN2,PCK1,REG1A,REG1B | 0.001728 |

**Supplementary Table 4. Molecular Functions (MF) and Cellular Component (CC) enrichment for 31 genes.**

| **Category** | **Term** | **Description** | **Symbols** | p-value |
| --- | --- | --- | --- | --- |
| Molecular Functions | GO:0042379 | chemokine receptor binding | DEFB4A,CXCL1,CXCL3 | 5.54E-05 |
| Molecular Functions | GO:0048018 | receptor ligand activity | DEFB4A,CXCL1,CXCL3,REG1A | 0.001481 |
| Molecular Functions | GO:0030546 | signaling receptor activator activity | DEFB4A,CXCL1,CXCL3,REG1A | 0.001572 |
| Molecular Functions | GO:0001664 | G protein-coupled receptor binding | DEFB4A,CXCL1,CXCL3 | 0.003374 |
| Molecular Functions | GO:0048306 | calcium-dependent protein binding | DMBT1,S100A8,S100A9 | 0.000101 |
| Molecular Functions | GO:0031406 | carboxylic acid binding | PCK1,S100A8,S100A9 | 0.000906 |
| Molecular Functions | GO:0046906 | tetrapyrrole binding | IDO1,TCN1,DUOX2 | 0.000476 |
| Cellular Components | GO:0034774 | secretory granule lumen | CXCL1,LCN2,S100A8,S100A9,TCN1 | 1.8E-05 |
| Cellular Components | GO:0060205 | cytoplasmic vesicle lumen | CXCL1,LCN2,S100A8,S100A9,TCN1 | 1.88E-05 |
| Cellular Components | GO:0042581 | specific granule | CXCL1,LCN2,TCN1 | 0.000586 |
| Cellular Components | GO:0031012 | extracellular matrix | DMBT1,PI3,S100A8,S100A9 | 0.002624 |
| Cellular Components | GO:0030312 | external encapsulating structure | DMBT1,PI3,S100A8,S100A9 | 0.00264 |
| Cellular Components | GO:0031252 | cell leading edge | REG1A,DUOX2,DUOXA2 | 0.009135 |
| Cellular Components | GO:0045177 | apical part of cell | ABCB1,DUOX2,DUOXA2 | 0.009979 |
| Cellular Components | GO:0030667 | secretory granule membrane | CD55,DMBT1,FCGR3B,VNN1,IDO1,PCK1 | 0.000277 |
| Cellular Components | GO:0031225 | anchored component of membrane | CD55,FCGR3B,VNN1 | 0.000699 |
